# The regulation of antiviral innate immunity through non-m^6^A RNA modifications

**DOI:** 10.3389/fimmu.2023.1286820

**Published:** 2023-10-17

**Authors:** Shenghai Shen, Li-Sheng Zhang

**Affiliations:** ^1^Division of Life Science, The Hong Kong University of Science and Technology (HKUST), Kowloon, Hong Kong SAR, China; ^2^Department of Chemistry, The Hong Kong University of Science and Technology (HKUST), Kowloon, Hong Kong SAR, China

**Keywords:** RNA modification, innate immunity, virus infection, 2’-O-methyltransferase, 5-methylcytidine, pseudouridine, RNA editing

## Abstract

The post-transcriptional RNA modifications impact the dynamic regulation of gene expression in diverse biological and physiological processes. Host RNA modifications play an indispensable role in regulating innate immune responses against virus infection in mammals. Meanwhile, the viral RNAs can be deposited with RNA modifications to interfere with the host immune responses. The *N*^6^-methyladenosine (m^6^A) has boosted the recent emergence of RNA epigenetics, due to its high abundance and a transcriptome-wide widespread distribution in mammalian cells, proven to impact antiviral innate immunity. However, the other types of RNA modifications are also involved in regulating antiviral responses, and the functional roles of these non-m^6^A RNA modifications have not been comprehensively summarized. In this *Review*, we conclude the regulatory roles of 2’-*O*-methylation (Nm), 5-methylcytidine (m^5^C), adenosine-inosine editing (A-to-I editing), pseudouridine (Ψ), *N*^1^-methyladenosine (m^1^A), *N*^7^-methylguanosine (m^7^G), *N*^6^,2’-*O*-dimethyladenosine (m^6^Am), and *N*^4^-acetylcytidine (ac^4^C) in antiviral innate immunity. We provide a systematic introduction to the biogenesis and functions of these non-m^6^A RNA modifications in viral RNA, host RNA, and during virus-host interactions, emphasizing the biological functions of RNA modification regulators in antiviral responses. Furthermore, we discussed the recent research progress in the development of antiviral drugs through non-m^6^A RNA modifications. Collectively, this *Review* conveys knowledge and inspiration to researchers in multiple disciplines, highlighting the challenges and future directions in RNA epitranscriptome, immunology, and virology.

## 1 Introduction

Numerous emerging research areas, including cancer immunotherapy, gene therapy, regenerative medicine, and the pandemic, underscore the significance of investigating the innate immune system for advancing human healthcare and pharmaceutical innovation (1–4). As the fields of biomedical and biotechnology forge ahead at an unprecedented pace, our comprehension of this intricate regulatory network continually evolves, with established paradigms being supplemented from different perspectives and interdisciplinary insights emerging.

As the first line of defense against invaders, the innate immune system protects the body from the harmful effects of viral infections. A typical antiviral response can be succinctly outlined as follows: first and foremost, the innate immune system recognizes virus pathogen-associated molecular patterns (PAMPs) and triggers downstream signaling pathways. PAMPs typically include viral single-stranded (ss) or double-stranded (ds) RNAs, dsDNAs, and viral proteins (5, 6). The molecules in the immune system that detect PAMPs and transmit signals are pattern recognition receptors (PRRs). Common types of RNA-sensing PRRs include Toll-like receptors (TLRs), Retinoic Acid Inducible Gene-I (RIG-I)-like receptors (RLRs), NOD-like receptors (NLRs), C-type lectin receptors (CLRs), Protein Kinase R (PKR), and 2’-5’-Oligoadenylate Synthetases (OAS) (5, 7). These PRRs are one of the main targets for RNA viruses to evade immune surveillance (8–11). The characteristics and functions of the major PRRs are described in **Table 1**. Upon activation of signaling pathways by PRR, a large number of cytokines and chemokines (Activator protein-1 (AP-1), interferon regulatory factors (IRFs), etc.) are induced through pathways like NF-κB and MAPK, initiating the expression of downstream immunomodulatory and antiviral genes, with notable examples such as interferon (IFN) family and interleukin (IL) family (15). Concurrently, macrophages, natural killer (NK) cells, granulocytes, dendritic cells (DCs), and other immune cells are activated, recruited, and dynamically engaged in the antiviral immune response (5).

**Table 1:** Brief description of PRR with RNA-sensing activity in mammalian cells.

PRR types	Brief description
TLR	There are 10 members in the human TLR family, which have structures consisting of leucine-rich domains for recognizing PAMPs and cytoplasmic domains for signal transduction. The subset involved in antiviral responses (TLR3, TLR7, TLR8, and TLR9) is located on the endosomal membrane to sense nucleic acids. TLR3 recognizes dsRNA, while TLR7 and TLR8 recognize ssRNA. Upon activation, TLRs utilize both MyD88-dependent and TRIF-dependent pathways to induce the synthesis of inflammatory cytokines and IFN-1 (12).
RLR	The RLR protein family is a cytoplasmic sensor that includes RIG-I, MDA5, and LGP2. They all contain a DExD/H box-containing RNA helicase domain for RNA binding and a CTD. The CTD of RIG-I is involved in recognizing the 5’-triphosphate of short panhandle dsRNA, while MDA5 tends to bind long dsRNA. They have also been shown to sense ssRNA. Upon activation, RIG-I and MDA5 utilize the N-terminal CARD to interact with MAVS on the mitochondria, which recruits TRAF family proteins or IKK to complete signal transduction. This leads to the upregulation of various inflammatory and transcription factors and the expression of ISGs. LGP2 does not have a CARD structure and may be involved in the regulation of MDA5 signaling (13).
PKR	PKR is an interferon-induced serine/threonine kinase that can be activated and autophosphorylated by cytoplasmic dsRNA or 5′-triphosphate-containing ssRNA. Activated PKR inhibits tRNA function by regulating eIF2a and thus affecting the expression of specific genes (14).

TRIF: Toll/IL-1 receptor domain-containing adaptor inducing interferon-beta; RIG-I: Retinoic acid-inducible gene I; MDA5: Melanoma differentiation-associated protein 5; LGP2: laboratory of genetics and physiology 2; CTD: C-terminal domain; CARD: Caspase recruitment domain; MAVS: Mitochondrial antiviral-signaling protein; TRAF: TNF receptor-associated factor; IKK: IκB kinase.

Due to the dynamic and rapid-response nature of the innate immune antiviral process, there has been considerable interest in exploring the realm of post-transcriptional regulation, a directly functional control layer of this process (16, 17). While extensive research has shed light on the roles of RNA splicing and non-coding (nc) RNA modulation in innate immunity regulation, there has been a growing focus on investigating the regulatory effects of RNA modifications (18–20). These chemical modifications have been demonstrated to be linked to the RNA sensing and activation of immune cells as early as 2005 (21). In recent years, many studies have reported diverse RNA modifications on viral and host RNAs and a complex network of interactions between RNA modifications and immune cells (22–25). Among these, *N*^6^-methyladenosine (m^6^A) stands out as the most abundant RNA modification in mammalian mRNA. The advancement of the base-resolution mapping tools targeting m^6^A, which are continually being refined, has facilitated the ongoing identification of its functional and mechanistic roles in diverse regulatory networks (26–32). Milestone studies continue to emerge, and the role of m^6^A in antiviral innate immunity regulation has been delving deeply, such as leading to immune evasion, influencing IFN production, and facilitating macrophage activation (33–35), with several related reviews published recently (34, 36).

Notably, in addition to m^6^A, many other RNA modifications have been demonstrated to possess a wide range of regulatory functions. Common types include 2’-*O*-Methylation (Nm), 5-methylcytidine (m^5^C), adenosine-inosine editing (A-to-I editing), pseudouridine (Ψ), *N*^1^-methyladenosine (m^1^A), *N*^7^-methylguanosine (m^7^G), *N*^6^,2’-*O*-dimethyladenosine (m^6^Am), and *N*^4^-acetylcytidine (ac^4^C). These non-m^6^A RNA modifications manifest diverse and intricate regulatory roles in controlling gene expression, modulating metabolic networks, and impacting the development of diseases (37), concomitant with the progress made in RNA epitranscriptome (38, 39). Intriguingly, these non-m^6^A RNA modifications are intimately linked with antiviral innate immunity, exerting biological and physicochemical regulatory functions upon both viral and host RNAs. Especially in recent years, the regulatory role of non-m^6^A RNA modifications in innate immunity has been heavily investigated, attracting considerable attention within translational medicine research (7, 20, 40).

Hence, in light of the present state of the cutting-edge investigation in non-m^6^A epitranscriptome and the gap of a summary related to innate immunity, here we comprehensively reviewed the regulatory role of the non-m^6^A RNA modifications in the antiviral innate immunity.

## 2 Non-m^6^A RNA modifications in antiviral innate immunity

**Figure 1:**
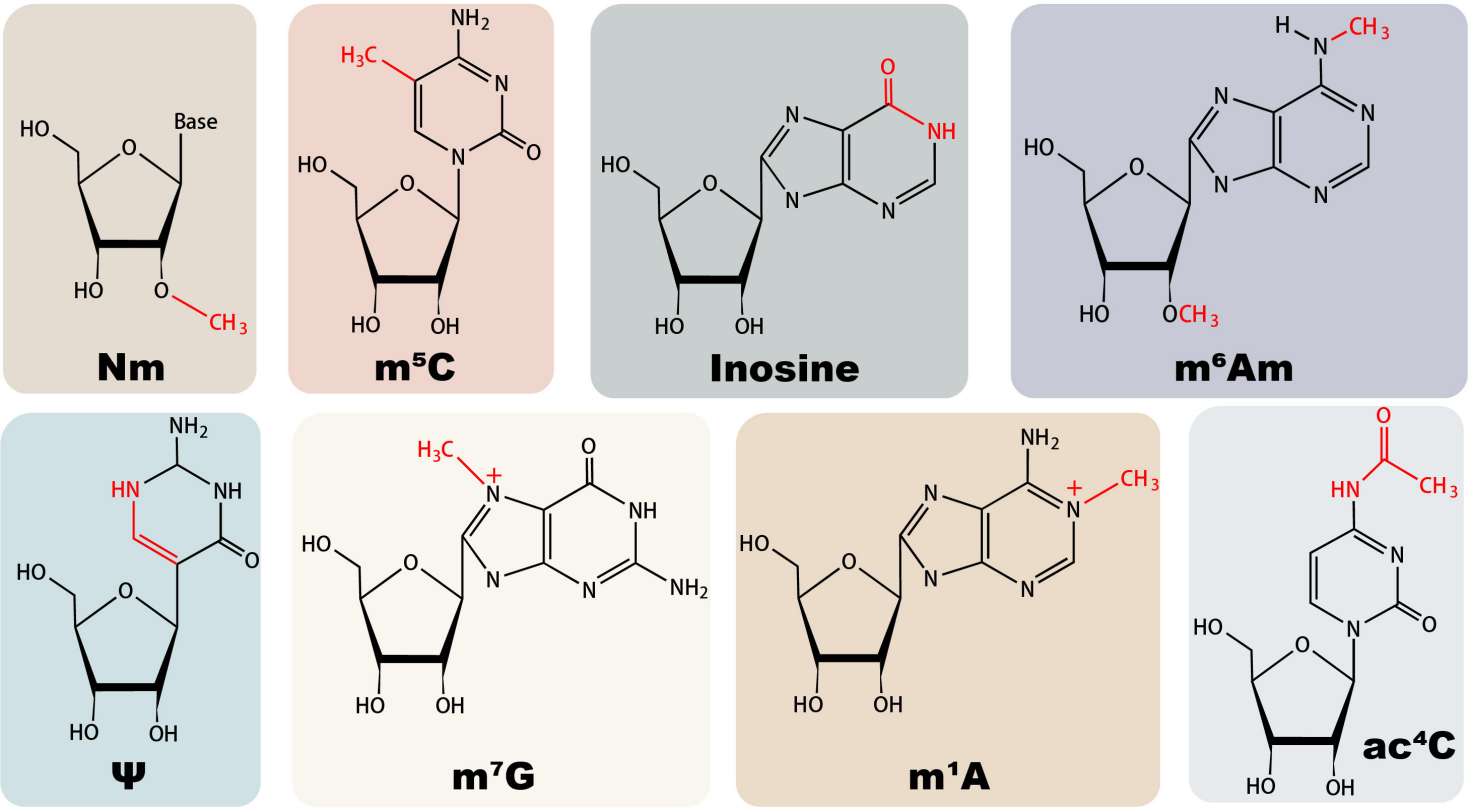
Structures of major non-m^6^A RNA modifications in mammals.

The role of diverse non-m^6^A RNA modifications in antiviral innate immune responses can be divided into the regulation of host RNA modification and viral RNA modification, impacting immune cell development and cytokine production, disguising endogenous RNA, and affecting viral development (36), with their chemical structures illustrated in **Figure 1**. From a dynamic perspective, the regulatory role of RNA modifications encompasses facilitating viral evasion from RNA sensing, infection-induced repercussions on cytokine production and signal transduction, and altering the epitranscriptome of immune cells to affect their function (37).

### 2.1 Regulatory role of 2’-*O*-methylation in antiviral innate immunity

2’-*O*-methylation (Nm) modification is a highly conserved modification in which the 2’-hydroxyl group of nucleotides is methylated by 2’-*O*-methyltransferase (2’-*O*-MTase) co-transcriptionally or post-transcriptionally. Discovered initially to exist in ribosomal RNA (rRNA) and transfer RNA (tRNA), Nm has since been found to exhibit varying levels of abundance in diverse RNA species (41). Base-resolution sequencing techniques have been developed to detect Nm modifications, bringing breakthroughs in understanding the stoichiometric characteristics of this modification (42, 43). In human mRNAs, Nm modification near the cap structure is generally added by Cap methyltransferase 1 (CMTR1) or CMTR2, while Nm within internal positions could be installed by Fibrillarin (FBL) and FTSJ3 regulators (25, 44, 45). The involvement of a ribonucleoprotein (snoRNP) complex containing a small nucleolar RNA of the C/D box family (snoRNA) is essential in FBL-regulated Nm modifications (46, 47). Besides maintaining stability and promoting translation, the cap Nm of human mRNA commonly serves as a molecular signature of endogenous host RNA, and the lack of this modification may lead to autoimmune diseases (48). Internal mRNA Nm can hinder translation elongation by disrupting the interaction between translation components and tRNA decoding efficiency (49, 50); meanwhile, it is reported to stabilize mRNA and enhance its expression capacity (47). Furthermore, Nm plays essential roles in other types of RNA, such as rRNA Nm affecting ribosome heterogeneity and stability, and tRNA Nm maintaining translation accuracy and tRNA stability (48, 51). Pathologically, Nm exerts a regulatory influence on the occurrence of diseases such as cancer, autoimmune diseases, and epilepsy (52–55).

**Figure 2:**
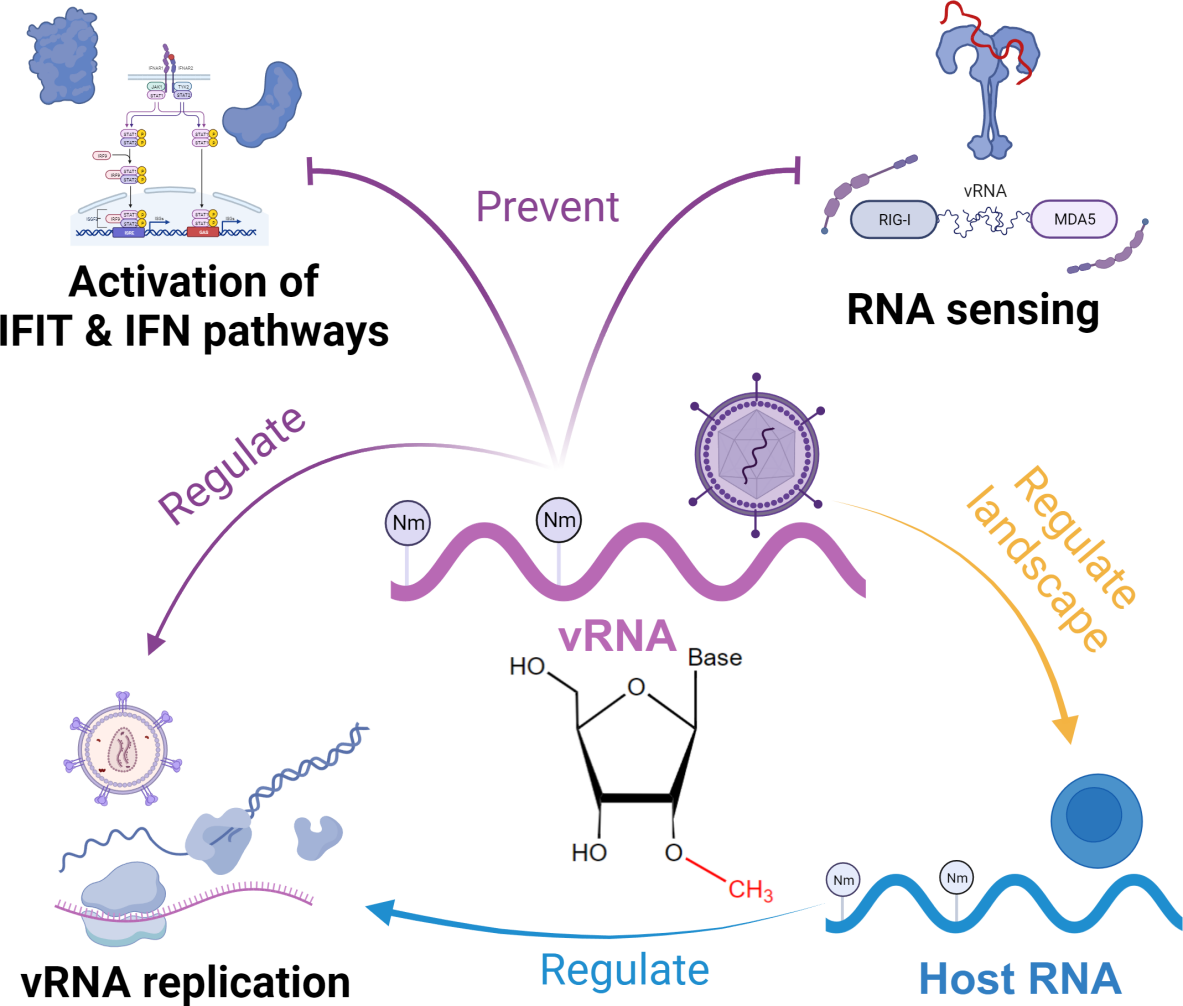
Functional roles of Nm in the regulation of antiviral innate immunity.

Apart from maintaining the stability of viral RNA by preventing the activity of host decapping and exoribonuclease protein, a paramount role of Cap Nm in antiviral innate immunity lies in facilitating viral RNA evasion from host RNA-sensing, thereby enabling immune evasion (**Figure 2**) (56). Since the Nm modification is installed at the first base (Cap 1 Nm) in eukaryotic host mRNA, numerous viruses employ this strategy to conceal themselves from detection by PRRs (57). By exhibiting Cap 1 Nm on their viral RNA, the viruses engage in a “subterfuge” that effectively evades recognition by RLRs, particularly RIG-I and MDA5 (58, 59), which disrupts signaling cascades, significantly suppresses the production of IFN, and impacting macrophage activation (60). In the case of RIG-I, this evasion effect can be attributed to steric hindrance resulting from the spatial obstruction caused by Nm when RIG-I binds to RNA, primarily facilitated by residue H830 (59, 61). On the other hand, although structural biology studies have inspired, the interaction mechanism between MDA5 and Nm still remains unclear. Recent studies suggest that Nm may reduce the catalytic activity switch of MDA5 (62). Moreover, viral RNA Cap 1 Nm also affects RNA sensing through TLR7 pathways, consequently impacting inflammatory responses (56). Although the inhibitory effect of Nm in bacterial tRNA on TLR activation has been studied to some extent, the relevant mechanisms and degree of inhibition in viruses are yet to be determined (63, 64).

Another escape strategy mediated by Cap Nm is to prevent the binding of viral RNA to IFN-induced protein with tetratricopeptide repeats 1 (IFIT1), an antiviral protein (65). IFIT1 is upregulated in response to viral infection, guided by interferon signals, and can recognize viral RNA lacking Cap Nm. It forms a complex with IFIT2 and IFIT3 and interacts with the eukaryotic initiation factor eIF3, inhibiting the transcription and translation of viral RNA, which is essential for IFN-induced antiviral response (60, 66). The internal RNA binding tunnel of IFIT1 exhibits a preference for RNA without Cap Nm, thus displaying a limited affinity for viral RNA with this modification (67). IFIT1B of the same family retains the ability to bind Cap1 Nm RNA, albeit with reduced affinity (68). Therefore, Cap Nm helps various viruses, such as West Nile virus, Zika virus, and human immunodeficiency virus (HIV), to escape the antiviral effect of IFN-induced antiviral factors (45, 69, 70). Coronaviruses lacking 2’-*O*-methyltransferase (2’-*O* MTase) exhibit high sensitivity to IFIT1 (71). Note that Cap Nm is only one of several strategies used by viruses to evade IFIT monitoring, while viral RNAs with Cap 1 Nm are still affected by IFIT1 at high concentrations; however, the viruses with only Cap 2 Nm are able to avoid IFIT1 effects completely, but this is rare among viruses (60, 67, 72, 73). Interestingly, a recent study reported that Cap 2 Nm in mammalian mRNAs shifts from Cap 1 Nm at a very slow methylation rate and mainly accumulates on host mRNAs, ensuring a low level of Cap 2 Nm in nascent viral RNAs and a certain level of immunostimulation (74).

Despite the incomplete comprehension of internal Nm modifications in viral RNA, recent studies have partially illuminated their regulatory role in innate immunity. Like internal Nm modifications in host mRNA, internal Nm modifications in viral RNA of various viruses can impair replication by affecting the elongation of the RNA polymerase (75). Interestingly, HIV is an exception to this. A recent report proved that internal Nm modifications induced by host 2’-O MTase promote HIV viral RNA replication (45). Furthermore, these modifications are implicated in immune evasion strategies employed by HIV, manifesting through their impact on MDA5 sensing and the antiviral activity of ISG20 (45, 76). While some investigations have shown that integrating Nm-modified adenosine into short RNAs can inhibit TLR7 activation, further exploration is warranted to determine if analogous mechanisms exist for other viral types (60, 77). Internal Nm modifications have been discovered in viral RNAs of many viruses, such as SARS-CoV-2, Ebola virus, and Dengue virus, but their functions remain unclear (75, 78, 79), awaiting more in-depth functional investigation.

In addition to the essential role in the development of immune cells such as macrophages and the expression of immune-related genes, host RNA Nm modification can also dynamically regulate the immune response through changes in Nm stoichiometry (80). The human Cap 1 2’-O MTase, CMTR1, exhibits an upregulated expression in response to interferon, thereby modifying the Nm status of specific antiviral ISG genes to enhance their expression and facilitate IFN-mediated antiviral response (25, 81). Some studies indicated the protective role of Nm modification on host RNA, such as increased vulnerability to viral infection in hosts lacking Nm-modified tRNA and resistance of Nm-modified host siRNA to targeting by poxviruses (82, 83). A recent study has also found that the introduction of Nm modifications on the RNA template significantly inhibits the synthesis of viral RNA (84). Moreover, viruses can manipulate the landscape of host RNA Nm modification. A typical example is that viruses can exploit FBL to modify host pre-rRNA’s Nm status, thereby attenuating protein synthesis. For instance, HIV infection disrupts FBL’s binding to nascent pre-rRNA, impairing ribosome biogenesis and function (85). Similarly, the Hendra virus orchestrates FBL methylation to influence proviral host genes and viral protein synthesis (44). Additionally, the disturbance of FBL has the potential to influence Nm modification level, consequently impeding the Type I IFN response and thereby facilitating viral infiltration into macrophages (86). Intriguingly, recent investigations have unveiled a decline in Nm sites within host mRNA upon SARS-CoV-2 infection, albeit the implications of this phenomenon on the host necessitate further study (78).

Comprehending how viruses synthesize Nm to circumvent immune responses is paramount in designing specialized antiviral medications. Three conventional ways for viral RNA to acquire Cap Nm have been identified, including utilizing host ‘writer’ enzymes (*e.g*., HIV), “cap snatching” from host mRNA (*e.g*., influenza virus), and encoding specialized enzymes and active sites for capping (*e.g*., SARS-COV-2) (25, 45, 87). The elucidation of these mechanisms propels advancements in translational medical research. Taking SARS-COV-2 as an example, several non-structural proteins (Nsps) are involved in viral RNA Nm modification, with pivotal participants including Nsp13, Nsp10, Nsp16, etc (88). Structural biology and biochemical studies of these 2’-*O*-MTases inspire drug development (89). Numerous drugs targeting these Nsp proteins have been conceptualized and developed (90–92). Similarly, drugs targeting the Nm synthesis mechanisms of viruses such as dengue, influenza, and Japanese encephalitis have also been reported (93–95). In addition, since viruses lacking the Cap Nm-deficient phenotype significantly attenuated virulence, these defective viruses demonstrate feasibility as a vaccine approach (96).

### 2.2 Regulatory role of m^5^C in antiviral innate immunity

RNA m^5^C is a modification that occurs at the 5th position of cytosine residues and is widely distributed in eukaryotes RNA, with high enrichment in tRNA and other non-coding RNAs (37). With the advancement of related high-throughput sequencing technologies, the regulatory mechanisms of m^5^C and the corresponding writer, eraser, and reader regulatory proteins have been continually explored (97–99). Although much remains unknown, m^5^C writers have been relatively more well-studied. Two classes of writers have been identified: the DNA methyltransferase 2 (DNMT2) and the NOL1/NOP2/SUN domain (NSUN) family (100). Notably, these eukaryotic m^5^C writers deposit this modification selectively based on RNA types. For instance, NSUN2 and NSUN6 are responsible for m^5^C deposition on mRNA, NSUN1 and NSUN5 modify cytoplasmic ribosomal RNA, and mitochondrial RNA m^5^C is installed by NSUN3 and NSUN4 (100). This complexity directly leads to the diverse regulatory functions of m^5^C in innate immunity. In host RNA, the typical role of m^5^C is to maintain RNA stability. Furthermore, m^5^C impacts mRNA export through the ‘reader’ protein ALYREF, safeguards tRNA against stress-induced damage, influences protein synthesis rates, and actively partakes in ribosome biogenesis (20, 37, 101). Besides its involvement in embryonic development processes, m^5^C is intimately linked to various diseases, such as cancer and neurological disorders (20, 101).

**Figure 3:**
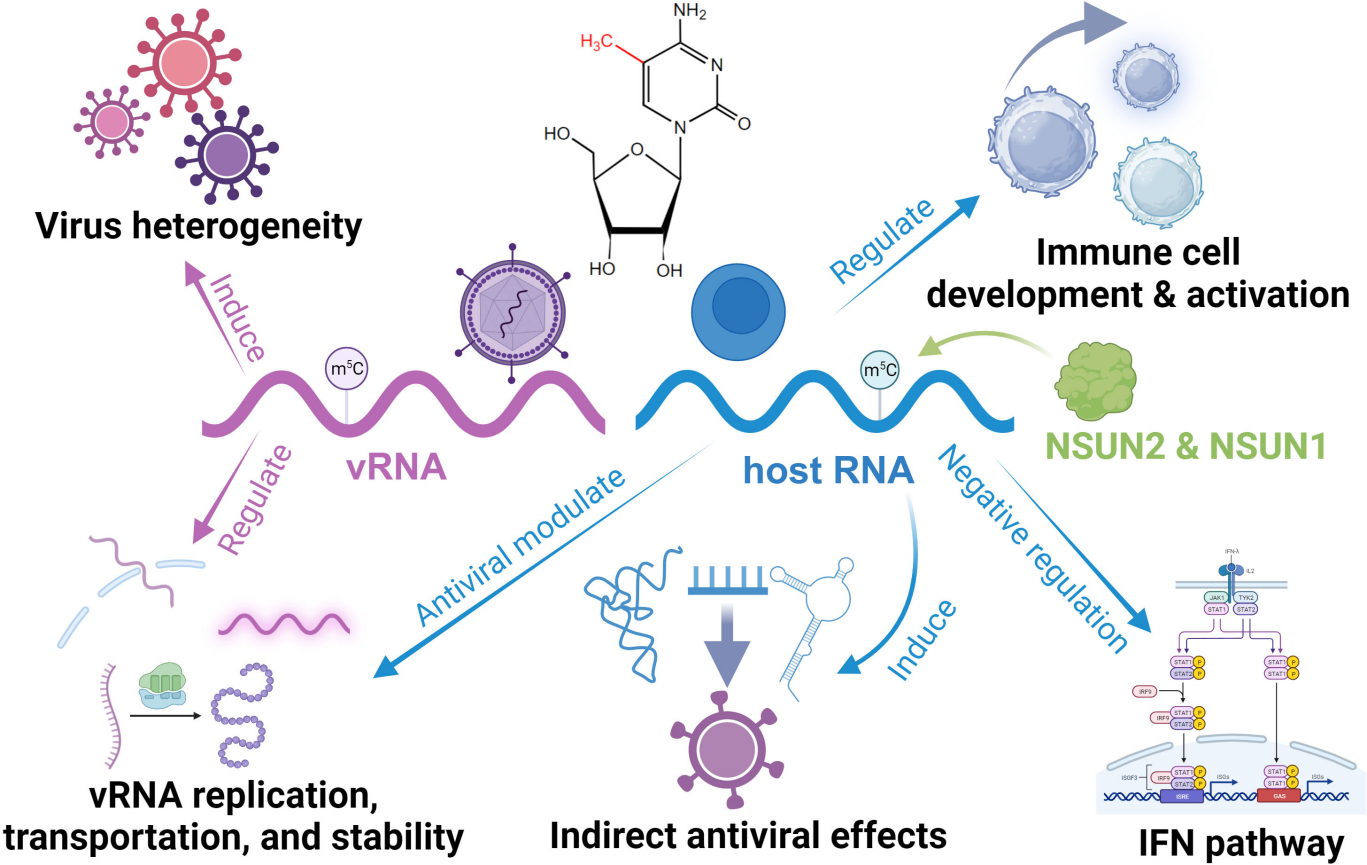
Functional roles of RNA m^5^C in the regulation of antiviral innate immunity.

Currently, there are no reports of viruses encoding their own m^5^C writer. However, they can utilize host writers to aid in their invasion (**Figure 3**). The most common case is the methylation of CpG islands on viral RNA mediated by DNMT2 and NSUN5 (102). Although the specific function is unclear, this may mediate viral heterogeneity (102). HIV has demonstrated its active recruitment of NSUN2 and DNMT for installing m^5^C on its viral RNA, thereby enhancing genome stability and facilitating replication, translation, and virus assembly efficiency (103, 104). Similar mechanisms have been observed in Mouse Leukemia Virus (MLV) and Alphaviruses, where a diminished NSUN2 level can reduce viral infection through downregulating m^5^C modifications (105, 106). Interestingly, the downregulation of NSUN2 in the Epstein-Barr (EB) virus increases viral RNA *in vivo*, as EB viral RNA degradation is m^5^C-dependent by RNase Angiogenin (107). In addition, viruses can employ m^5^C modifications on viral RNA to influence nuclear export and infectivity via interaction with host m^5^C readers like ALYREF (108, 109). Some studies have also reported the enrichment of m^5^C in the viral genome following infection; however, the specific function awaits further exploration (110).

In host cells, m^5^C is essential for maintaining immune homeostasis and immune cells like CD4 T Cells development (111, 112). In the innate antiviral response, m^5^C and its regulator have a sophisticated role. NSUN5 can bind to viral RNA and enhance the RNA-sensing function of RIG-I, functioning as a cardinal receptor (113). NSUN6 is involved in plasma cell formation (114). Meanwhile, DNMT2 responds to infection and relies on the dynamic installation of m^5^C to regulate the expression of antiviral genes, facilitating an efficient response (115, 116). NSUN1 inhibits HIV-1 replication and prolongs its latency by installing m^5^C on its transactivation response element RNA (117). It is worth mentioning that NSUN2, the most reported m^5^C writer associated with antiviral activity, exhibits functionally diverse regulatory roles. In response to viral infection, NSUN2 downregulates the levels of specific ncRNAs and alters their m^5^C levels, which directly and indirectly modulates the type I interferon response mediated by the RIG-I signaling pathway and enhances the antiviral response (118). In addition, NSUN2 can convert vault RNA (vtRNA) into smaller fragments through m^5^C installation (119, 120). Some viruses can induce high vtRNA expression, inhibiting the activation of PKR and subsequent IFN response, silencing the host antiviral immune response (121). Similarly, tRNA-derived non-coding fragments (tRFs) generated with the involvement of NSUN2 can be utilized by viruses for their immune escape with viral RNA replication (122–124). In addition, NSUN2 may interact with uridylated deaminase, which inhibits viral activity (102).

Although there are currently rare antiviral drugs targeting RNA m^5^C, due to substantial overlap with regulators of DNA 5-methylcytosine, a large number of antiviral drugs (e.g., azacytidine and decitabine) that target DNA methylation also exhibit effects on RNA m^5^C (125). Nonetheless, given that the dearth of regulatory mechanisms has only been filled in the last few years, the controversial distribution on the viral genome, and the bidirectional regulatory properties of the regulator in innate immunity, present a formidable challenge to the advancement of drugs targeting m^5^C (126).

### 2.3 Regulatory role of A-to-I editing in antiviral innate immunity

A-to-I editing is an irreversible modification that widely exists in pre-mRNA, mRNA, and ncRNA, converting the amino group at position C_6_ of adenosine to a carbonyl group, resulting in inosine (37). In mammals, this modification is mediated by adenosine deaminases acting on RNA (ADARs) and adenosine deaminase acting on tRNA (ADAT), and these proteins have multiple dsRNA binding domains and a catalytic center (127). There are three types of ADARs in humans: ADAR1, ADAR2, and ADAR3 (which lacks catalytic activity *in vitro* and is considered a negative regulator of editing) (128). Among them, ADAR1 is responsible for the vast majority of modifications. Most ADAR’s actions occur within double-stranded RNAs formed by inverted *Alu* repeat elements scattered throughout the genome (128). This modification can disrupt RNA secondary structure and destabilize dsRNA. In the bioprocess, ADARs are involved in pre-mRNA processing (129). In host mRNA, inosine in non-coding regions can regulate specific gene expressions by adding or subtracting splicing donor or acceptor sites thus affecting alternative splicing, creating or destroying miRNA binding sites, and affecting mRNA stability (129–131). Within coding regions, A-to-I editing can regulate translation efficiency and generate new protein products (132). In ncRNA, ADARs play a role in the biogenesis of miRNA and circular RNA, and they can also adjust the miRNA targets (133). In addition, ADAR can change the secondary structure of lncRNA, thereby affecting its interaction with miRNA (20). Pathologically, A-to-I editing has been found to be associated with cancer, neurodegenerative diseases, and autoimmune diseases (127, 134, 135).

**Figure 4:**
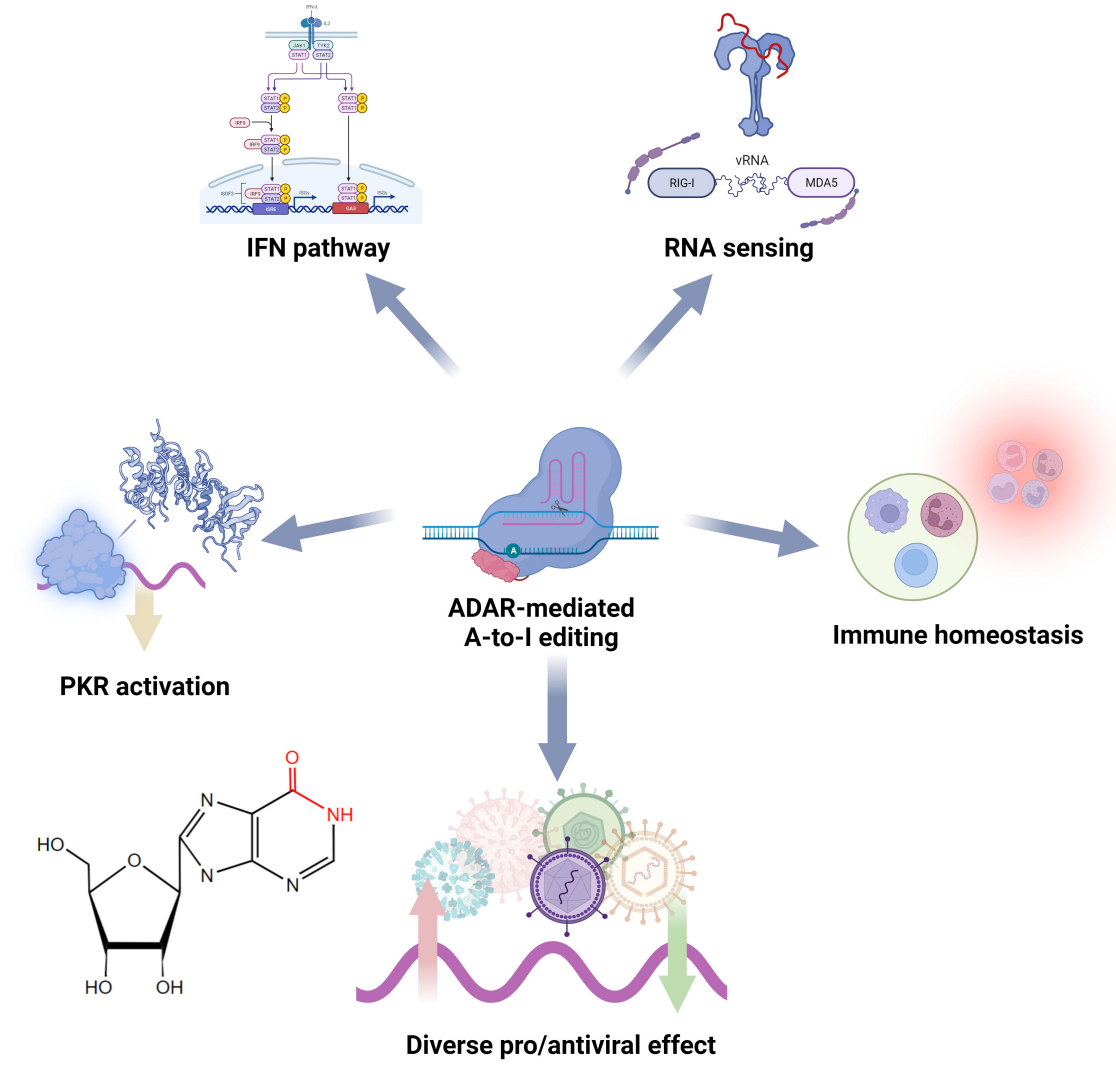
Functional roles of ADAR and A-to-I editing in the regulation of antiviral innate immunity.

In host cells, ADAR1, especially its IFN-responsive isoform ADAR1 p150, is a critical IFN inhibitory factor in antiviral innate immunity, and its negative regulation is essential for suppressing abnormal antiviral responses and maintaining immune homeostasis (**Figure 4**) (136). Endogenous dsRNA may induce innate immune activation and cause autoimmune inflammatory diseases. ADAR1 possesses the ability to modify these immunogenic molecules through A-to-I editing, thereby influencing their interaction with dsRNA PRRs and effectively thwarting downstream sensor (e.g., MAVS) activation as well as ISG-IFN and pathways and inflammation (128). IFN signaling requires down-regulation of ADAR1-p110 during viral infection to execute effective antiviral activity (137). These mechanisms are also essential to prevent excessive immune responses to the viral RNA (138). Mutations in ADAR can be observed in many autoimmune diseases, such as Aicardi-Goutières syndrome (AGS) (139). In the RLR pathway, ADAR1 probably influences MDA5’s binding affinity to dsRNA by inducing structural alterations through installed inosine residues and imposing limitations on RIG-I RNA-sensing capacity via protein-protein interaction since this inhibition is not dependent on catalytic activity but rather RNA binding activity (140, 141). In the PKR pathway, ADAR1 inhibits the autophosphorylation activation of this dsRNA PRR, thereby preventing subsequent eIF2α-induced translation arrest (142). Some studies have also shown that ADAR affects the activation of the OAS-RNase L pathway and its induced RNA degradation, autophagy, and cell apoptosis (143). Moreover, ADAR1 has been found to interact with Z-DNA binding protein 1, limiting self-Z-RNA sensing and avoiding the type I IFN pathway (144, 145). Other studies demonstrated that ADAR1 can modulate immunity by directly editing IFN pathway components (128, 146, 147). Note that the details of how these editings affect PRRs and sensors need to be further investigated. In addition, host A-to-I editing regulates the development and activation of immune cells. A-to-I editing is essential for maintaining intracellular homeostasis in DCs and macrophages, and the loss of ADAR1 leads to cellular metabolic disorders (148). Furthermore, ADAR can affect macrophage polarization by inhibiting the biogenesis of miR-21 (147).

Through editing the viral RNA, transcripts, template strands, and immune-responsive RNA, ADAR’s impact on viruses can be either proviral, antiviral, or even have no effect. This complexity primarily depends on the combination of the virus and host and the specificity of the editing sites. Like Nm, viruses can utilize/recruit host ADAR to edit viral RNA, effectively disguising it as “self” to evade detection by RNA-sensing (149). Notable examples include HIV, hepatitis C virus (HCV), Ebola virus, and measles virus (MeV), which utilize ADAR1 to edit their genomes/transcriptomes, thereby evading detection by RLR and other PRRs, and inhibiting IFN induction by suppressing IRF3 activation, thus blocking the immune response (150–154). Furthermore, some viruses have shown the ability to induce high expression of ADAR1 to raise the threshold for immune response (155). In addition, Dengue viruses were shown to modulate ADAR abundance by regulating the expression of microRNAs targeting ADAR1 (156). Different subtypes of ADAR1 (p150 and p110) may possess distinct modification sites and functions within the same virus. For instance, during influenza A virus infection, p150 hinders RLR signaling and assumes a pro-viral role; conversely, p110 appears to exert an antiviral effect by editing viral RNA to impact replication efficiency (157–160). On the other hand, ADAR2 has also been shown to promote the immune escape of the Borna disease virus by editing its genome (161). Interestingly, the linear correlation between PKR and IFN-β protein levels, as well as the antagonistic effect of PKR on ADAR’s immune inhibitory function in various viruses, suggests a balanced interaction between PKR and ADAR1 in antiviral immunity (154, 162). Escape mechanisms utilizing non-structural proteins and the transactivation response element were identified in viral infections by regulating PKR, interacting with the PKR activating compound of translation, and manipulating ADAR1 to inactivate PKR, which inhibited the suppression of viral replication and the subsequent formation of antiviral stress granules (163–167). Intriguingly, this “ loophole “ in ADAR that viruses can exploit appears to be an evolutionary invention for preventing aberrant antiviral responses (160, 168, 169). This is also evidenced by its complex regulatory network and low concentration level in the cytoplasm (170).

ADAR can achieve its proviral effects through other mechanisms as well. Viruses can exploit ADAR and influence viral RNA’s replication, transcription, and protein production efficiency through editing (171). A typical example is HIV, which hijacks ADAR1 and ADAR2 to achieve A-to-I editing, resulting in faster translation efficiency and significantly enhanced release of progeny viruses (152, 172). A study has demonstrated that ADAR1 is essential for efficiently replicating HIV-1 in T cells (173). It is worth noting that the stimulatory effect of ADAR1 on HIV-1 is achieved through both editing-dependent and editing-independent mechanisms (153). Moreover, ADAR1 can modify viral protein products, thereby impacting viral proliferation and infectivity. For instance, by modifying the RNA template of the hepatitis delta virus, it can induce the production of proteins that foster viral particle packaging (174, 175). ADAR can also provide a replication environment for viruses by disrupting endogenous RNA interference (176). In addition, ADAR1 has been shown to interact with the DICER protein and inhibit its cleavage of edited dsRNA (177). Viruses may recruit ADAR to suppress DICER, thereby escaping viral gene silencing (128). Recent studies have also found that ADAR editing accelerates the evolution of SARS-CoV-2 in humans and may be related to the infectivity of its spike protein (175, 178).

As an immune regulator, ADAR has been shown to have antiviral activity. Cells lacking ADAR exhibit increased susceptibility to various viruses, while overexpression of ADAR1 can inhibit viral replication (179). During different stages of viral infection, ADAR exhibits different proviral and antiviral effects that depend on the inflammatory response (180). Similar to its proviral regulatory, ADAR can also exert antiviral effects through site-specific editing. For instance, by editing HCV’s replicon, ADAR significantly curtails its replication; by editing MeV’s non-encapsidated defective interfering RNA, it diminishes infectivity; by editing long terminal repeat retrotransposons, it restricts their activity (181–183). ADAR can also reduce the expression of the encephalomyocarditis virus-encoded circRNA for antagonizing PKR activation by editing it to promote immune activation (184, 185). Additionally, ADAR1 can upregulate antiviral microRNA expression in response to infection (141). An interesting case is that since PKR activity favors HCV replication, the inhibitory effect of ADAR on PKR indirectly suppresses HCV (186). Furthermore, many studies have shown that, in the long run, the evolutionary effects of ADAR-induced editing pressure on viral genomes are likely detrimental (168, 187, 188). ADAR editing of the SARS-CoV-2 genome may reduce transmissibility (189). Thus, ADAR may be an “evolutionary weapon”. ADAR has also demonstrated an association with antiviral immune responses in many other cases, but whether ADAR directly involves these modulations remains unclear (186).

ADAR has been widely used in RNA editing therapy for diseases like cancer and metabolic disorders, as it exerts regulatory influence devoid of genome disruption (190). However, within the domain of antiviral drug development, despite the potential utility of investigating ADAR’s editing of viral genomes to inspire the development of potent vaccines, studies of related drugs are still sparse (191, 192). Numerous enigmas remain shrouded in ambiguity. Firstly, the mechanisms and biological effects of ADAR’s editing of viral genomes in the immune response are still inconclusive, and these effects may vary among different viruses. Viruses can also induce editing of host RNA during infection, such as the reduction of A-to-I editing of endogenous *Alu* RNA caused by SARS-CoV-2 (193). Further research is needed on the role of ADAR in virus-host interactions. Secondly, understanding how ADAR rapidly responds to viral infections, how host cells dynamically regulate the concentration of ADAR, and how this “double-edged sword” affects the diverse impact on dsRNAs are crucial for drug design. Research from a molecular evolution perspective may inspire. Molecular evolution studies may furnish valuable insights in this regard. Finally, ADAR has been shown to interact with other RNA modification regulators such as m^6^A readers and affect non-viral responsive RNAs like long interspersed element 1 (194, 195). Thus, more research perspectives with ADAR at the center of the immune regulatory network are essential for comprehending its antiviral prowess.

### 2.4 Regulatory role of Ψ in antiviral innate immunity

In addition to the earlier mentioned A-to-I editing, some other RNA editing modifications also contribute to the innate immune response. Ψ is the most abundant and widely distributed cellular RNA modification known as the “fifth nucleotide” (196). Ψ is the C5-glycoside isomer of uridine, and this conserved modification appears irreversible. The most notable function of Ψ is maintaining various RNAs’ structure and stability (197). This gives an understanding of the responsiveness of Ψ to stress (198). tRNA exhibits the most abundant Ψ sites, and these modifications are crucial for translation (198). In mRNA, Ψ affects pre-mRNA splicing and impact mRNA stability. One representative function is that when Ψ modification occurs at the stop codon, it inhibits translation termination of the mRNA (37). In addition, Ψ plays a role in small nuclear RNAs (snRNAs), in maintaining their structures and regulating RNA-protein interactions (199, 200). Research on the regulators of Ψ modification is still ongoing, and various writers (members in pseudouridine synthase) have been identified, but there have been no reports of erasers and readers. Recently, the quantitative sequencing technologies have been developed for mapping Ψ transcriptome-wide, which have advanced the study of this abundant modification’s role in biological processes and diseases context (201, 202).

**Figure 5:**
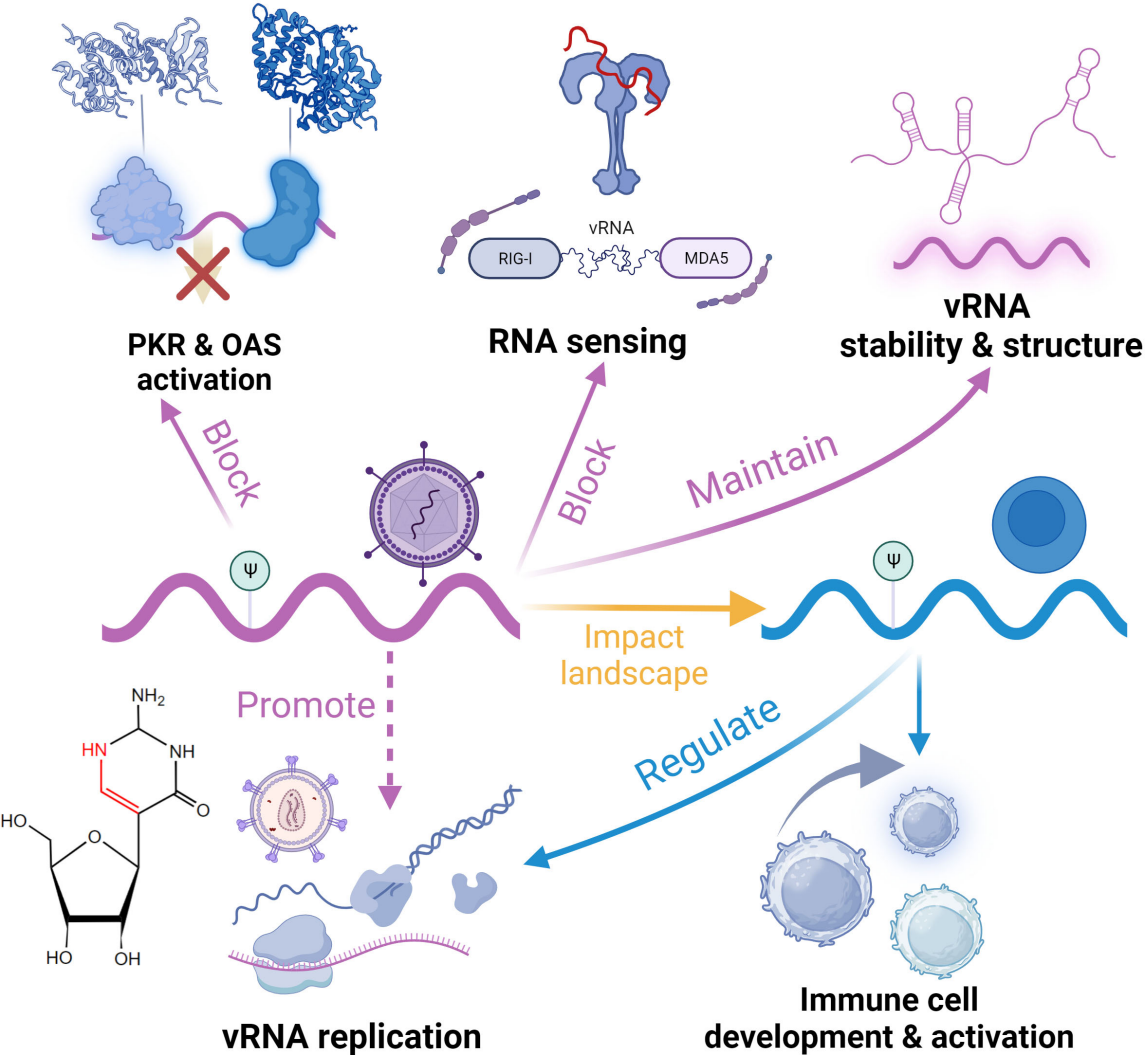
Functional roles of pseudouridine (Ψ) in the regulation of antiviral innate immunity.

Ψ has been detected in the genomes of many viruses, such as SARS-CoV-2 and influenza viruses (203, 204). Dated back to 2010, it was reported that Ψ on RNA can inhibit the activation of PKR and avoid degradation (**Figure 5**) (205). In viruses, Ψ modifications can help viral RNA escape detection by host PRRs. Studies have revealed that although RIG-I can discern RNA with Ψ, it cannot induce conformational changes that initiate downstream signaling (206). Moreover, Ψ has been demonstrated to obstruct the activation of TLRs, particularly TLR7 and TLR8 (21). Additionally, Ψ has also been reported to diminish the activity of OAS, thereby enabling modified RNA to be translated for an extended duration (207). Although many cases suggest the regulatory role of Ψ in antiviral RNA sensing, the specific mechanisms of immune evasion are still unclear. Considering the enhancing effect of Ψ on mRNA translation efficiency, Ψ on viral RNA also seems to promote viral gene expression; however, validation *in vivo* is lacking. Besides maintaining immune homeostasis, host RNA Ψ modification also plays a responsive role in antiviral immunity. A typical example is HIV-1 infection. The initiation of HIV reverse transcription is highly dependent on Ψ modifications on host tRNA, which stabilize the complex formed between tRNA and viral RNA (208). Furthermore, Ψ on 7SK snRNA regulates its stability and structure, influencing the formation of super elongation complexes during infection and indirectly inhibiting HIV-1 transcription. The loss of this modification promotes HIV-1 escape from latency and facilitates reverse transcription (209). In addition to affecting the development of immune cells, host Ψ has been shown to influence the activation of DCs and CD8+ T cells (21). Interestingly, many studies have observed an elevation in the abundance of host Ψ following viral infection, suggesting a combination of effects of host antiviral response and viral actions (204, 210). This may regulate antiviral gene expression by regulating RNA splicing and mRNA stability (204). A recent study also found that this stoichiometric regulation of Ψ was induced by the IFN pathway (211). Regardless, Ψ plays a role in host-virus interactions in the antiviral response, and the mechanisms need further investigation. In antiviral therapy, the most representative application of Ψ is to reduce immune response and improve translation efficiency in mRNA vaccine (7). This technology has been applied to commercialized vaccines (e.g. SARS-COV-2 vaccine) (212, 213).

### 2.5 Regulatory role of m^7^G, m^1^A, and m^6^Am in antiviral innate immunity

Other forms of non-m^6^A RNA methylation also exert influence on the innate immune response. m^7^G, prevalent *N*^7^-methylated guanosine found in eukaryotic RNA, holds significant importance. Analogous to Nm, m^7^G serves as an integral component of the cap structure within eukaryotic RNA polymerase II (pol II) transcripts and is distributed internally across mRNA, tRNA, and rRNA (214). Cap m^7^C is a classic eukaryotic RNA structure that stabilizes transcripts and prevents degradation. Additionally, this structure regulates bioprocesses such as mRNA splicing, nuclear export, transcription elongation, and translation (215–218). Despite its late recognition as a crucial modification, numerous studies have demonstrated its regulatory role in mRNA translation efficiency, and related regulators have been continuously discovered, with quantitative sequencing techniques being developed (219–221). Similar to cap Nm, viruses can acquire cap m^7^G through various mechanisms. Common mechanisms include utilizing host Pol II to synthesize RNA with cap m^7^G, stealing cap from short host mRNA, and encoding their own capping methyltransferase (87). Cap m^7^G plays a crucial role in maintaining the stability of viral RNA, and notably, it helps viral RNA escape the action of 5’exonucleases and avoid degradation (215). In addition, cap m^7^G also promotes the translation and expression of viral RNA (222). These regulatory mechanisms for viral RNA cap m^7^G are highly analogous to those in the host mRNAs. Interestingly, the previously mentioned cap-snatching mechanism not only prevents the expression of host RNA but can also lead to gene fusion with the host, resulting in the production of immunomodulatory chimeric proteins after viral infection (223). Furthermore, a study reported hypermethylation of HIV-1’s Cap m^7^G directly affects infectivity (224). Compared to Cap Nm, the virus’s Cap m^7^G does not seem to promote its escape from RNA sensing recognition by RLR and other PRRs (61). As for internal m^7^G, it has not been reported to be found in viral RNA, and it is still unclear whether the landscape of the host internal m^7^G is responsive to viral infection. Antiviral drugs that interfere with viral RNA capping have been extensively developed based on the capping mechanisms of different viruses (225).

m^1^A, methylation of the N1 position of adenosine, is an abundant and conserved modification in eukaryotic non-coding RNAs. In recent years, we have witnessed the development of multiple single-base resolution sequencing technologies, which have sought to address the challenge of mapping m^1^A modifications transcriptome-wide (226–228). The m^1^A modification introduces steric hindrance, which affects base pairing and the spatial conformation of RNA. It also influences the interactions between RNA and proteins, other RNAs, or small molecules (37). These diverse functions are closely related to the location of m^1^A. In addition to being highly abundant in tRNA (affecting stability, translation efficiency, and accuracy) and rRNA (maintaining ribosome function), m^1^A has also been found to be distributed in mRNA (229). The functions of m^1^A on host mRNA are still poorly understood, although its site-specific involvement in translation regulation has been identified (228, 230). Interestingly, m^1^A is closely related to m^6^A. In addition to their convertibility to m^6^A, they share a variety of regulators like fat mass and obesity-associated (FTO) protein as an eraser (231, 232). In viral RNA, m^1^A has been identified and enriched in specific regions (203). However, its involvement in innate immunity is not yet clear. Viral infection has been shown to impact the expression levels of m^1^A writers and erasers on host mRNA, and some of them may have proviral effects (203). Intriguingly, upregulated m^1^A on host RNA inhibits the activity of the replication complex of SARS-COV-2 and thus achieves an antiviral effect, an effect that does not appear to be related to the steric hindrance caused by m^1^A (233). As for the impact on immune cells, research on m^1^A has mainly focused on cancer, such as its influence on immune cell infiltration (111). Nevertheless, this modification demonstrates research value in antiviral innate immunity. Further studies need to be conducted, especially on the mechanisms of regulators, the responsiveness of these modifications to viral infection, and the interactions with m^6^A during the antiviral response.

m^6^Am is another well-known mRNA modification, typically occurring at the first nucleotide after the cap structure in the 5’ UTR of eukaryotic mRNA, known as the Cap Nm position. When this nucleotide is adenosine, the phosphorylated CTD-interacting factor 1 (PCIF1) converts Am to m^6^Am (37). Similar to m^6^A and m^1^A, FTO mediates the demethylation of m^6^Am (234). The detailed role of m^6^Am in regulating mRNA metabolism is still under investigation. Existing studies have demonstrated its impact on mRNA stability and translation, but the conclusions from these studies are contradictory (235–237). m^6^Am plays a regulatory role in antiviral immune processes. Like Nm, m^6^Am installed by host PCIF1 on viral RNA can mediate immune evasion. The presence of m^6^Am on viral RNA prevents its detection by ISGs and weakens the antiviral effects of the IFN-β pathway (238). Unlike RIG-I or IFIT1 sensing, this effect does not depend on RIG-I or IFIT1 sensing. Additionally, viral m^6^Am can prevent viral RNA degradation by nucleases (234). Host m^6^Am also undergoes dynamic changes in antiviral responses, exhibiting both antiviral and proviral effects. HIV infection induces ubiquitination and degradation of host PCIF1, leading to a decrease in m^6^Am on cellular mRNA and regulating the host transcription factor ETS proto-oncogene 1 (ETS1) to promote viral replication (239). In this process, PCIF1 exerts its antiviral function by affecting ETS1 stability by installing m^6^Am. On the other hand, in SARS-CoV-2 infection, PCIF1 maintains the stability of angiotensin-converting enzyme 2 (ACE2) and transmembrane serine protease 2 (TMPRSS2) mRNA by installing m^6^Am, promoting their expression and facilitating viral infection (240). In summary, m^6^Am, as a typical cap modification, has gained attention since the development of single-base resolution sequencing techniques (241). However, further research is needed to explore its functional connections and differences with Nm, as well as the role of PCIF1 in viral infection.

### 2.6 Regulatory role of ac^4^C and other RNA modifications in antiviral innate immunity

In addition to the regulatory modifications described above, some less-studied RNA modifications also function in antivirals. Although there is limited research on ac^4^C, this newly discovered, unique acetylation modification in eukaryotes has a vital role in bioprocesses and immune response. Initially discovered and demonstrated to regulate ribosome maturation and protein translation ability in tRNA and rRNA, ac^4^C was later found to be distributed in mRNA and enriched in the coding sequence (CDS) region using antibody-based sequencing methods (242). On mRNA, ac^4^C in the CDS region can maintain stability and promote translation, while the 5’ UTR has been found to potentially inhibit translation initiation by affecting the interaction between mRNA and tRNA/ribosomes (243, 244). ac^4^C has been found to be associated with diseases such as cancer, neurodegenerative disorders, and inflammation (242). In viral infections, the presence of ac^4^C on various viral RNAs plays a regulatory role. HIV-1 can utilize the host ac^4^C writer *N*-acetyltransferase 10 (NAT10) to add ac^4^C to viral RNA, increasing its stability and promoting replication (245). Inhibiting NAT10 can suppress the spread of HIV-1 without affecting cell viability. Interestingly, another investigation reported that NAT10 actually fosters HIV-1 latency while impeding Tat-mediated transcriptional processes (246). Further research is needed. NAT10 has also been found to recruit and add ac^4^C on viral RNA in Enterovirus, specifically recruiting host proteins to enhance viral RNA stability and translation (247). Additionally, changes in the abundance of ac^4^C in the 5’ UTR region of specific genes in the host have been observed after infection with the influenza A virus, which may be related to the virus-induced expression of NAT10 and its pro-viral effects (203, 248). Nevertheless, the specific functions of this modification distributed in the genomes of diverse viruses and the role of NAT10 in antiviral innate immunity await further investigation.

There are also several other low-abundance modifications detected on viral RNA, such as 5,2’-*O*-dimethylcytosine (m^5^Cm) (60). However, the functions of these modifications are still unknown. In addition, a number of other types of RNA editing also play a role in the regulation of antiviral innate immunity, although it is inconclusive whether these edits are categorized as RNA modifications. An example is the addition of uridine to the 3’ RNA terminus catalyzed by terminal uridyltransferases (TUTases), also known as uridylation and poly(U) tails. This editing occurs on almost all classes of RNAs and regulates processes such as mRNA decay, histone expression, and miRNA metabolism and targeting (249). Uridylation has been shown to have regulatory functions in innate immunity. TUTases can directly edit the genomes of various viruses, forming a “poly(U) tag” effect to promote immune response by mediating RNA exosome degradation, facilitating viral RNA decay, and targeting viral proteins for antiviral purposes (250, 251). Loss of TUTases leads to an increase in viral mRNA and protein levels. Furthermore, TUTases can be activated by TLR and regulate mRNA stability to promote the production of various cytokines, participating in immune responses (252). Another type of RNA editing involved in antiviral innate immunity is C-to-U deamidation editing mediated by the apolipoprotein B mRNA editing catalytic polypeptide-like (APOBEC) protein family. APOBEC proteins can significantly inhibit the replication of various retroviruses, endogenous viruses, and DNA viruses, including HIV-1, human T-cell leukemia virus type 1, hepatitis B virus, etc. (253). This viral restriction effect is mediated by multiple mechanisms, including inhibition of viral infection factor expression, disruption of viral particle assembly, excision of viral RNA bases, and direct editing of the genome of DNA viruses (253, 254). Moreover, APOBEC-mediated editing is essential for immune cell differentiation, development, and function (255–257). Many reviews have summarized the regulatory effects of APOBEC-mediated editing, so we will not review them in this work.

## 3 Conclusion and perspective

In this *Review*, we summarized the regulatory roles of eight typical non-m^6^A RNA modifications in antiviral innate immunity within the current scope of knowledge, including 2’-*O*-methylation (Nm), 5-methylcytidine (m^5^C), adenosine-inosine editing (A-to-I editing), pseudouridine (Ψ), *N*^1^-methyladenosine (m^1^A), *N*^7^-methylguanosine (m^7^G), *N*^6^,2’-*O*-dimethyladenosine (m^6^Am), and *N*^4^-acetylcytidine (ac^4^C). It is evident that compared to the extensively studied m^6^A modification, there is still a significant gap regarding the immune regulatory functions of non-m^6^A RNA modifications. The lack of clarity regarding the regulators of non-m^6^A RNA modifications has caused gaps in understanding the mechanisms underlying innate immune responses, particularly the utilization and induction patterns of viruses. In addition, low abundance, complexity of distribution, and diversity of responsive effects are all challenges for the study of non-m^6^A RNA modifications in antiviral immunity.

Considering the recently emerging base-resolution sequencing methods, the quantitative analysis of non-m^6^A RNA modifications, such as Ψ, Nm, m^7^G, m^5^C, m1A, and ac4C, has been enabled to resolve the challenges in studying the location and stoichiometry of these modifications on viral and host RNA, as well as the regulatory modes of RNA modification landscapes during host-virus interactions. These recent advances in base-resolution sequencing technology offer the super-resolution profiles of non-m^6^A RNA modifications transcriptome-wide; meanwhile, the breakthroughs in quantitative mapping tools could aid the comprehensive investigation of non-m^6^A RNA modifications in antiviral innate immunity through monitoring the dynamics of these RNA modifications.

Besides, some non-m^6^A RNA modifications have been demonstrated to facilitate the immune escape and replication of certain viruses; however, these non-m^6^A RNA modifications are also essential for the host to enhance the antiviral immune response. Thus, the manipulation of RNA modifications at specific sites within host RNA or viral RNA may have an impact on the development of antiviral drugs with therapeutic potential. Overall, the functional and mechanistic study of RNA modifications in antiviral innate immunity has led to entirely new perspectives, yielding in-depth insights into translational medicine and potentially benefiting related research in biological sciences, biomedical engineering, clinics, and the pharmaceutical industry.

## Author contributions

SS: Conceptualization, Investigation, Methodology, Writing – original draft, Writing – review & editing. LZ: Conceptualization, Funding acquisition, Project administration, Resources, Supervision, Writing – review & editing.
